# An investigation of codon usage pattern analysis in pancreatitis associated genes

**DOI:** 10.1186/s12863-022-01089-z

**Published:** 2022-11-25

**Authors:** Yuanyang Li, Rekha Khandia, Marios Papadakis, Athanasios Alexiou, Alexander Nikolaevich Simonov, Azmat Ali Khan

**Affiliations:** 1 Third-Grade Pharmacological Laboratory On Chinese Medicine Approved By State Administration of Traditional Chinese Medicine, Medical College of China Three Gorges, Yichang, China; 2 College of Medical Science, China Three Gorges University, Yichang, China; 3 Department of Biochemistry and Genetics, Barkatullah University, Bhopal, MP 462026 India; 4 Department of Surgery II, University Hospital Witten-Herdecke, University of Witten-Herdecke, Heusnerstrasse 40, 42283 Wuppertal, Germany; 5 Department of Science and Engineering, Novel Global Community Educational Foundation, Hebersham, Australia; 6 AFNP Med Austria, Vienna, Austria; 7 Stavropol State Agrarian University, Stavropol, Russia; 8 Pharmaceutical Biotechnology Laboratory, Department of Pharmaceutical Chemistry, College of Pharmacy, King Saud University, Riyadh, 11451 Saudi Arabia

**Keywords:** Pancreatitis, RSCU, Nucleotide skew, Codon correlation, Compositional constraints

## Abstract

**Background:**

Pancreatitis is an inflammatory disorder resulting from the autoactivation of trypsinogen in the pancreas. The genetic basis of the disease is an old phenomenon, and evidence is accumulating for the involvement of synonymous/non-synonymous codon variants in disease initiation and progression.

**Results:**

The present study envisaged a panel of 26 genes involved in pancreatitis for their codon choices, compositional analysis, relative dinucleotide frequency, nucleotide disproportion, protein physical properties, gene expression, codon bias, and interrelated of all these factors. In this set of genes, gene length was positively correlated with nucleotide skews and codon usage bias. Codon usage of any gene is dependent upon its AT and GC component; however, AGG, CGT, and CGA encoding for Arg, TCG for Ser, GTC for Val, and CCA for Pro were independent of nucleotide compositions. In addition, Codon GTC showed a correlation with protein properties, isoelectric point, instability index, and frequency of basic amino acids. We also investigated the effect of various evolutionary forces in shaping the codon usage choices of genes.

**Conclusions:**

This study will enable us to gain insight into the molecular signatures associated with the disease that might help identify more potential genes contributing to enhanced risk for pancreatitis. All the genes associated with pancreatitis are generally associated with physiological function, and mutations causing loss of function, over or under expression leads to an ailment. Therefore, the present study attempts to envisage the molecular signature in a group of genes that lead to pancreatitis in case of malfunction.

**Supplementary Information:**

The online version contains supplementary material available at 10.1186/s12863-022-01089-z.

## Background

Pancreatitis refers to an inflammatory disorder that affects the pancreas, usually accompanied by abdominal pain. It damages the pancreas to varying degrees and the adjacent and distal organs and results in elevated serum pancreatic enzymes. Pancreatitis could be acute or chronic, with common clinical outcomes and shared etiological and genetic risk factors. Risk factors include gallstones, tobacco smoke, alcohol abuse, hypertriglyceridemia, etc. [1]. The pancreas secretes various enzymes, including trypsin, chymotrypsin, elastase, and carboxypeptidase. In the pancreas, digestive enzymes are secreted in inactivated form, and these become activated in the duodenum. The intestinal transmembrane protease enteropeptidase activates trypsinogen to trypsin, which finally activates chymotrypsinogens, proelastases, and procarboxypeptidases into their active form. Trypsinogen has a unique property of auto-activation and happening inside the pancreas results in inflammatory disorder pancreatitis. As a mode of defence, a serine protease inhibitor Kazal type 1 (*SPINK1*) is secreted to prevent the auto-activation of trypsinogen. In the *SPINK1* gene, a mutation is found as a risk factor for chronic pancreatitis. Few other relevant genes associated with enhanced risk factors are Serine Protease 1 (*PRSS1)*, a gene related to hereditary pancreatitis, *CFTR*, *CTRC,* Carboxypeptidase A1 *(CPA1), PRSS1, *and *SPINK1 *enhance the pancreatitis risk by promoting harmful trypsinogen activation or impaired trypsinogen degradation and/or trypsin inhibition [2, 3]. Other genetic factors related to pancreatitis are Calcium Sensing Receptor (*CASR),* Claudin *2 (CLDN2),* Carboxyl Ester Lipase *(CEL),* Cathepsin B (*CTSB),* Myosin IXB *(MYO9B),* Ubiquitin Protein Ligase E3 Component N-Recognin 1 *(UBR1)*, and Fucosyltransferase 2 (*FUT2)* [1]. Mutations in *PRSS1, SPINK1, CTRC, CASR*, and *CFTR* were linked with pancreatitis and pancreatic cancers when the molecular basis of pancreatitis was investigated. The most vital risk factors linked with genetic variations in *PRSS1, SPINK1,* CF Transmembrane Conductance Regulator *(CFTR)*, and to a lesser extent, Chymotrypsin C (*CTRC)* and *CASR* [4]. *SPINK1* mutations are a stronger risk factor in cases of chronic pancreatitis associated with recurrent trypsin activation [5]. The elements that are involved in intra-pancreatic activation of trypsinogen regulation mechanism include polymorphism or mutations in genes *CTRC, CASR*, Trypsinogen gene (*PRSS1*, 2 and 3), *CTSB*, *SPINK1* and *CFTR* [6]. Among half of the idiopathic chronic pancreatitis patients, the role of genetic alteration in *PRSS1, SPINK1, CTRC*, and *CFTR* genes was identified. There is accumulating evidence of the involvement of genetic risk factors in pancreatitis and associated pathologies, suggesting the importance of genetic elements in pancreatitis [7]. There are 64 codons present in the standard genetic code that encodes for 20 amino acids. Excluding three stops codons and methionine and tryptophan, encoded by single codons, all other amino acids are encoded by two or more than two codons. Such codons are called synonymous codons. All the synonymous codons are not used equally. Thus, there is a bias in the usage of synonymous codons considered codon usage bias (CUB) that varies among species, organs [8], and tissue [9] types. Codon usage is a complex phenomenon and influenced by compositional constraints [10], amino acid frequency [11], physical properties of the protein [12], tRNA abundance [13], hydrophobic nature of the protein [13], gene length [14], temperature [15], protein structure [16], etc. Evolutionary forces like translational selection and mutational forces also influence codon usage [17]. Since the synonymous codons are the codons encoded for the same amino acid, these were previously considered to pose no impact on the resultant protein. However, these synonymous variants have a significant impact on protein expression. For example, in the gene, von Willebrand Factor (VWF) that cleaves hemostatic protease ADAM Metallopeptidase with Thrombospondin Type 1 Motif 13 (*ADAMTS13*), effects of synonymous mutations have been investigated, and it was found that not only the non-synonymous but the synonymous variants also influence mRNA and protein expression, conformation, and function [18]. Furthermore, bioinformatics tools establish the relationship between mRNA stability, relative synonymous codon usage (RSCU), and intracellular protein expression. It was found that synonymous variants substantially impact the above-mentioned properties [18]. mFold and KineFold are the secondary structure predictors of changes in minimum free energies of the mRNA fragments containing synonymous variants and help determine altered protein expression levels, attributed to alternative mRNA splicing and /or changes in mRNA structure/folding minimum free energy [19].

Synonymous single nucleotide variants (sSNV) are a participant in various disorders like pulmonary sarcoidosis, attention-deficit/hyperactivity disorder, and cancer [20]. In addition, synonymous variants in 4 genes [(Cadherin Related 23 (*CDH23),* SLC9A3 Regulator 1 *(SLC9A3R1),* Rhomboid Domain Containing 2 *(RHBDD2)*, and Inter-Alpha-Trypsin Inhibitor Heavy Chain 2 (*ITIH2*)] linked with alzheimer's disease warrant comprehensive scrutiny of genetic variations [21]. Among sSNV, codon bias is also a factor, where one particular codon is preferred over the other. Pancreatitis is an inflammatory disease that severely affects lifestyle and quality of life. The genetic factors are responsible for the development of pancreatitis, but so far, no work has been conducted related to codon usage patterns of these genes, so we became anxious to know the pattern of codon usage choices and use of synonymous variants in the genes involved in pancreatitis to investigate the molecular patterns present in genes. In the present study, we investigated 26 genes that are supposed to have roles in developing pancreatitis.

The present study will help identify various factors associated with synonymous codon bias, including nucleotide disproportion, dinucleotide proportions, gene expression, and effects of mutational, compositional, and selection forces in shaping the codon usage of genes. Codon usage analysis provides insight into the gene or genome evolution and adaptation of various environmental conditions. It also provides knowledge about the expressivity of genes [22]. Furthermore, it also provides meaningful information regarding genomic architecture [23]. The present study will also help understand the specific molecular signatures related to the gene set. The information regarding the overexpressed and underexpressed codons provide information for constructing synthetic gene for altered expression and gene augmentation.

## Results

### Compositional analysis

**Fig. 1:**
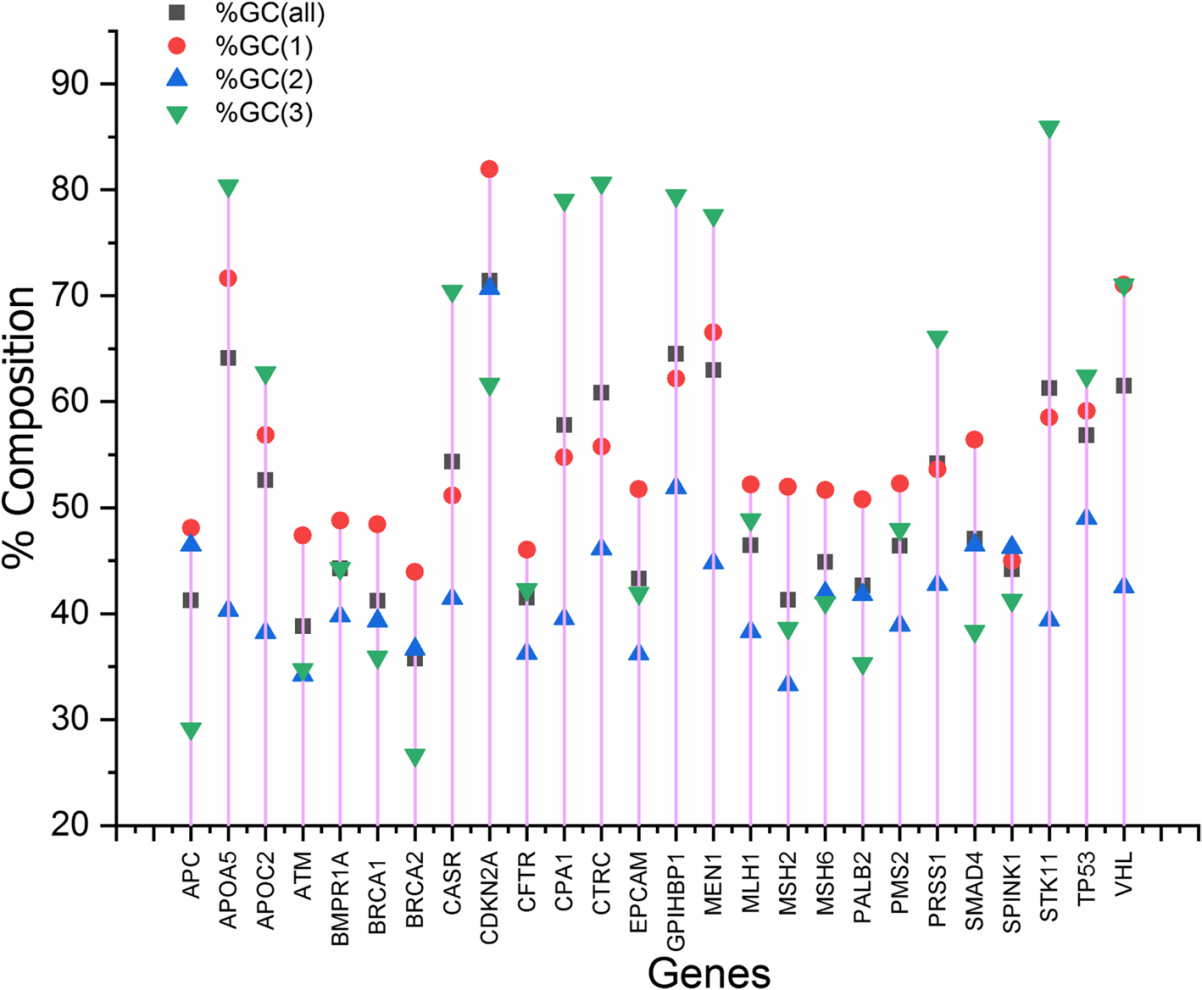
Stem diagram for GC composition for all the 26 genes involved in pancreatitis. In a few genes, %GC3 was highest, while in a few %GC1 was highest. Color code for each GC composition at different codon positions is given inside the figure

The composition generally affects the codon usage bias [24]. Geometric mean-based composition of nucleotides at various codon positions was observed, and it was observed that %T occurrence was the least (22.00%) among all the four nucleotides. In comparison, %A and %G were almost equal (25.99% and 25.63%, respectively). The minimum variance was observed for %C2 (10.86), while the maximum was for %C3 (132.98). Standard deviation was maximum for %C3 (11.53) while the minimum for %C2 (3.29). %AT composition was a little less (49.17%) than %GC (50.82%) composition. Percent GC3 composition at an overall level and all the three codon positions are given in Fig. 1. Mean %GC3 and %GC1 are approximately equal in percent composition (54.73% and 54.20%, respectively), while %GC2 composition was the least (mean value 43.49). A positive GC skew shows the richness of G over C, and the negative GC skew represents the richness of C over G [25]. GC skew values were 1.54, 2.09, 0.24 for GC1, GC2, and GC3, respectively. The skew values were positive for %GC components at all three codon positions. It is suggestive of the dominance of G over C at all three codon positions. However, the extent was different. At the GC3 position, the G to C bias was the maximum.

### Dinucleotide odds ratio

The dinucleotide odds ratio depicted that the dinucleotide CpG, TpA, and, GpT are underrepresented (in 81%, 58%, and 62% genes, respectively). At the same time, ApA, ApG, CpA, GpA, and TpG are overrepresented in more than 50% of pancreatitis-associated genes (50%, 65%, 54%, 50%, and 50%, respectively). Rest other dinucleotides are randomly used. The odds ratio for individual genes depicted that though the CpG dinucleotide is underrepresented in the maximum of genes, it was overrepresented in two genes Von Hippel-Lindau Tumor Suppressor *(VHL)* and cyclin-dependent kinase inhibitor 2A (*CDKN2A)*. CpT, GpA and TpG dinucleotides were the nucleotide underrepresented in none of the genes. Similarly, ApC, GpT, TpA and TpC were the nucleotides overrepresented in none of the genes. Dinucleotides ApT, CpG, GpT, TpA, and TpT were underrepresented (52.04%, 73.46%, 61.22%, 90.81% and 69.38% of genes, respectively) while ApG, CpA, CpC, GpC, GpG and TpG were over represented in more than 50% of housekeeping genes (57.14%, 63.26%, 54.08%, 52.04%, 61.22% and 62.64% respectively).

### RSCU analysis

**Fig. 2:**
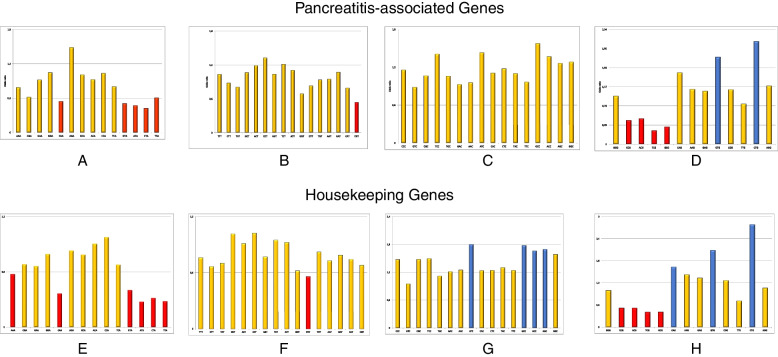
Depiction of RSCU values in pancreatitis associated genes: **A** A ending codons; **B** T ending codons; **C** C ending codons; **D** G ending codons. Depiction of RSCU values in Housekeeping genes: **E** A ending codons; **F** T ending codons; **G** C ending codons; **H** G ending codons. Orange bars show random usage, while red and blue bars show underrepresentation and overrepresentation of codons, respectively

RSCU analysis of 26 genes associated with pancreatitis showed a preference for G/C ending codons. However, amongst G/C ending codons CCG, ACG, TCG, and GCG were the codons that were underrepresented despite being CG ending codons (Fig. 2). GCC, CAG and GTG were the codons that were either overrepresented or randomly presented in 26 genes studied and underrepresented in none of the pancreatitis associated genes. When the RSCU values of individual codons were observed, it was seen that CTG and GTG codons were over-represented. GTA, ATA, CTA, TTA, CGT, CCG, ACG, TCG, GCG are the codons containing CpG and TpA dinucleotides, that were underrepresented. Codon CAA is the only codon underrepresented and does not contain CpG or TpA dinucleotide.

CGT is underrepresented in the pancreatitis gene set, while in housekeeping genes, GTT is underrepresented among T-ending codons. All C ending codons are randomly used in pancreatitis, while in housekeeping genes, ATC, GCC, ACC, and AGC codons are overrepresented, and other codons are randomly used. G ending codons showed a similar pattern for pancreatitis-associated genes and housekeeping genes except for codon CAG, which is overrepresented in pancreatitis genes while randomly presented in housekeeping genes. Here the difference in codon usage between pancreatitis and housekeeping gene is evident (Fig. 2).

### Comparison of Pancreatitis associated genes’ codon usage with housekeeping genes’ codon usage

To elucidate whether pancreatitis-associated genes display distinct features than any other gene set, we compared codon usage of pancreatitis-associated gene set with codon usage of the housekeeping gene set. For comparison, we performed variance analysis, PCA analysis, and comparative analysis of rare and frequent codons between the two gene sets.

**Fig. 3:**
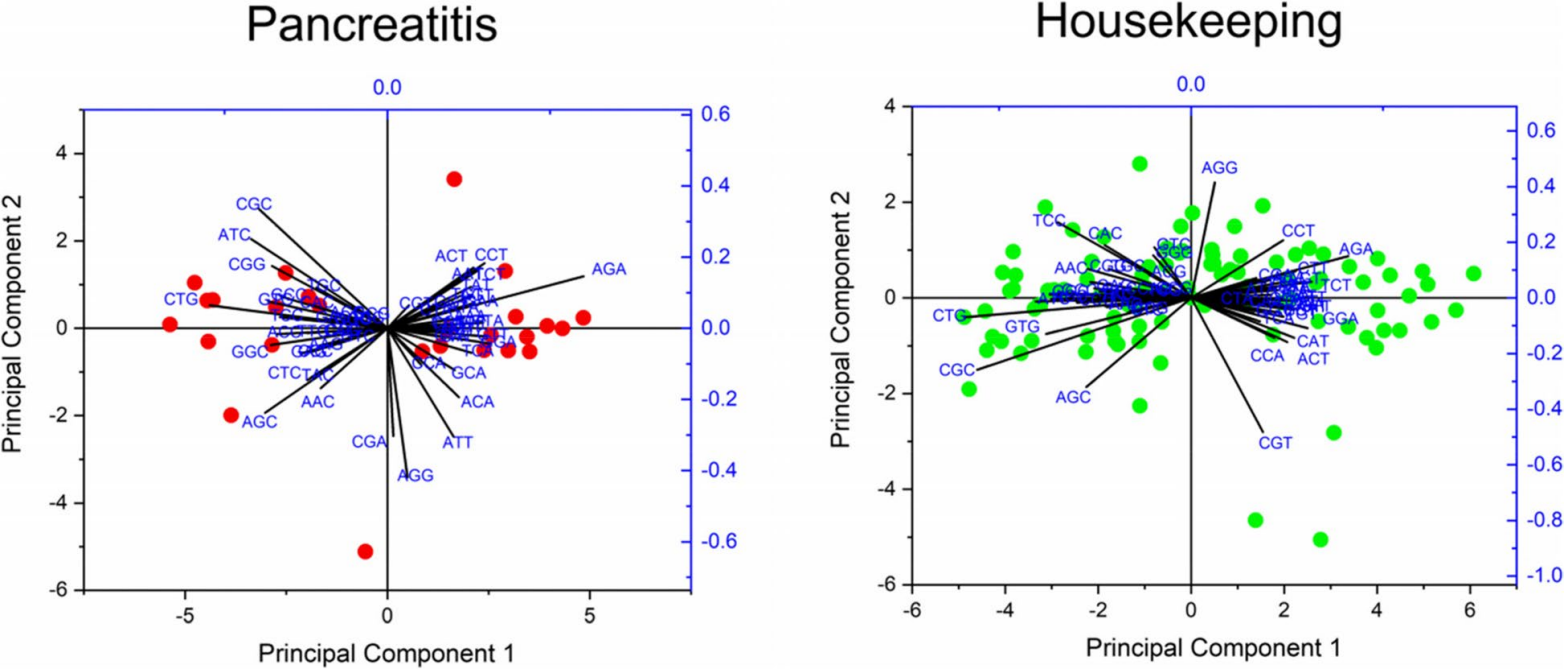
PCA for Pancreatitis associated genes. Analyses reveal the maximum contribution of AGG, CGC, ATT, and CGA codons in variation of CUB. Red dots show the positions of pancreatitis-associated genes across the axes. PCA for housekeeping associated genes reveals the maximum contribution of CGT, AGG, AGC, and CTG codons in the variation of CUB. Green dots show the positions of housekeeping genes across the axes

**Fig. 4:**
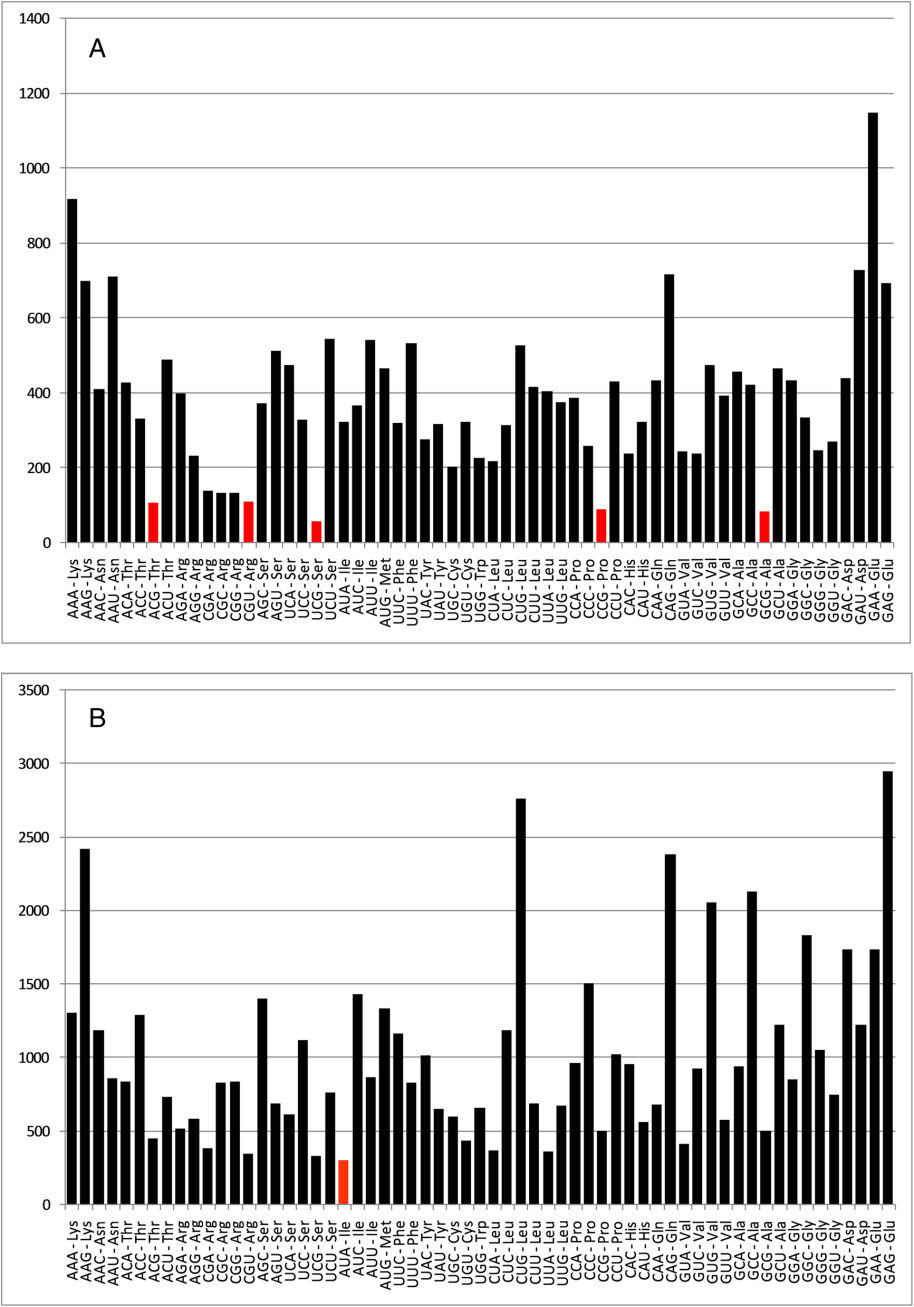
**A **Codons ACG-Thr, CGT-Arg, TCG-Ser, CCG-Pro, and GCG-Ala are rare in pancreatitis-associated genes. **B **Codons ATA- Ileu is rare in housekeeping genes. The Y-axis indicates the frequency of codons, while X-axis is indicative of various codons. Threshold ≤ 0.5% is set for rare codons which are depicted by red bars

- *Comparison of codon usage*
  Kolmogorov–Smirnov test is performed to compare two samples when two populations can be different [26]. We performed the test using PAST4.10 software with 1000 permutations. The results are presented in Table 1. Of 59 codons, 32 were statistically different in pancreatitis and housekeeping gene set.
- *Comparison of most influencing codons affecting CUB of pancreatitis and housekeeping gene sets*
  The PCA analysis was performed based on the RSCU values of codons of genes involved in pancreatitis. PCA analysis revealed that PC1 contributed 54.09% while PC2 contributed 9.51% variation in pancreatitis associated genes. Most genes were present near the X-axis, revealing that CUB is not much variable. Only two genes, *APOC2* and *SPINK1* showed different codon biases based on the RSCU values. A biplot analysis revealed that codons AGG, CGC, ATT, and CGA exhibited maximum loading values across the first two maximum contributing PCs (loading values 0.419, 0.3359, 0.305, and -0.302, respectively), suggestive that these codons are contributing maximum to the codon bias in pancreatitis associated genes (Fig. 3).
  To investigate whether the codon usage pattern is unique to the pancreatitis-associated gene set, we compared pancreatitis-associated genes' codon usage pattern with the housekeeping gene set encompassing 98 genes. The housekeeping gene set displayed a different codon usage pattern than pancreatitis-associated genes. PC1 (Principal component 1) and PC2 contributed 44.05% and 5.62% variation, respectively. Codons CGT, AGG, AGC, and CTG contributed maximum (loading values -0.452, 0.415, 0.332, and 0.290, respectively) towards codon usage bias across the first two maximum contributing PCs. Based on our comparative studies between pancreatitis-associated and housekeeping gene sets, it is evident that the codon usage pattern is distinct in the pancreatitis-associated gene set.
- *Comparative analysis of rare and frequent codons*
  In both gene sets, we compared the occurrence of rare codons (occurrence ≤ 0.5%). For this purpose, we determined the frequency of codons per thousand and plotted it as Fig. 4. Frequency of one codon for housekeeping genes (AUA-Ile) (Fig. 4A) and five codons for pancreatitis associated genes (ACG-Thr, CGT-Arg, TCG-Ser, CCG-Pro, GCG-Ala) (Fig. 4B) were found below threshold 0.5%. The results indicated that both gene sets use different rare codons. In the pancreatitis-associated gene set, the GAA-GAA codon pair (Gly-Gly) was most frequent (*n* = 84), while 647 codons pairs were absent. In the housekeeping gene set GAG-GAG codon pair (Glu-Glu) was the most abundant codon pair (*n* = 240), while 366 codon pairs were absent.

**Table 1:** Comparison of variance between average RSCU values of the pancreatitis gene set and housekeeping gene set

Codons	Average RSCU of HK gene set (*n* = 100)	Average RSCU of Pancreatitis gene set (*n* = 26)	*p* value	Level of significance	Codons	Average RSCU of HK gene set (*n* = 98)	Average RSCU of Pancreatitis gene set (*n* = 26)	*p* value	Level of significance
TTT	0.759	1.064	0.008	^**^	GCC	1.773	1.522	0.088	NS
TTC	1.241	0.936	0.007	^**^	GCA	0.781	1.024	0.010	^*^
TTA	0.275	0.627	0.446	NS	GCG	0.422	0.324	0.049	^*^
TTG	0.713	0.868	0.046	^*^	TAT	0.710	0.791	0.365	NS
CTT	0.658	0.901	0.014	^*^	TAC	1.290	1.133	0.199	NS
CTC	1.254	1.166	0.176	NS	CAT	0.750	0.804	0.510	NS
CTA	0.321	0.443	0.094	NS	CAC	1.230	1.042	0.119	NS
CTG	2.780	1.995	0.004	^**^	CAA	0.362	0.572	0.008	^**^
ATT	0.924	1.171	0.025	^*^	CAG	1.638	1.428	0.015	^*^
ATC	1.805	1.333	0.011	^*^	AAT	0.724	0.952	0.022	^*^
ATA	0.271	0.495	0.223	NS	AAC	1.256	0.971	0.009	^**^
GTT	0.558	0.873	0.003	^**^	AAA	0.577	0.831	0.007	^**^
GTC	0.944	0.829	0.221	NS	AAG	1.423	1.092	0.001	^**^
GTA	0.405	0.548	0.158	NS	GAT	0.787	1.065	0.006	^**^
GTG	2.093	1.750	0.005	^**^	GAC	1.213	0.935	0.004	^**^
TCT	0.946	1.194	0.030	^*^	GAA	0.666	0.945	0.002	^**^
TCC	1.508	1.269	0.040	^*^	GAG	1.334	1.055	0.001	^**^
TCA	0.668	0.857	0.041	^*^	TGT	0.837	0.907	0.323	NS
TCG	0.411	0.243	0.004	^**^	TGC	1.103	1.016	0.278	NS
AGT	0.768	1.077	0.062	NS	CGT	0.677	0.561	0.959	NS
AGC	1.700	1.359	0.015	^*^	CGC	1.462	1.041	0.039	^*^
CCT	1.028	1.304	0.017	^*^	CGA	0.673	0.736	0.636	NS
CCC	1.474	1.142	0.018	^*^	CGG	1.283	1.016	0.090	NS
CCA	0.970	1.077	0.360	NS	AGA	0.833	1.466	0.060	NS
CCG	0.528	0.478	0.424	NS	AGG	1.073	1.180	0.859	NS
ACT	0.906	1.198	0.039	^*^	GGT	0.619	0.671	0.205	NS
ACC	1.670	1.334	0.032	^*^	GGC	1.591	1.276	0.004	^**^
ACA	0.897	1.002	0.182	NS	GGA	0.788	1.116	0.280	NS
ACG	0.528	0.466	0.348	NS	GGG	1.001	0.936	0.148	NS
GCT	1.025	1.130	0.201	NS	–	–	–	–	–

^***^p < 0.001

^**^p < .01

^*^p < 0.05, *NS* non significant

### Association of gene length with nucleotide disproportion

**Fig. 5:**
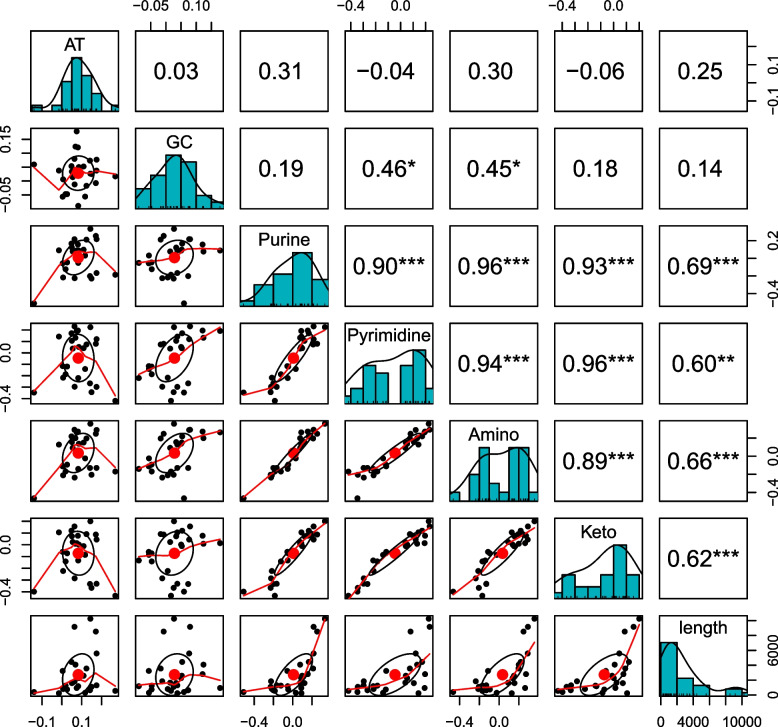
Matrix plot showing the correlation between the compositional skews and length. Black triangles are the compositional features of the genes, while red line indicates the regression line. The upper right matrix is showing the correlation coefficient

To investigate whether the gene length can affect the nucleotide skew, we calculated the six nucleotide skews i.e., AT skews, GC skews, purine skew, pyrimidine skew, keto skew, and amino skews. Its association with gene length was determined through correlation analysis. The length was found to be positively correlated with purine skew (*r* = 0.685, *p* < 0.001) pyrimidine skew (*r* = 0.601, *p* < 0.01) keto skew (*r* = 0.659, *p* < 0.001) and amino skews (*r* = 0.620, *p* < 0.001) for pancreatitis associated genes. The correlation plot between the skews and gene length is given in Fig. 5. We did a correlation analysis between housekeeping gene length and nucleotide disproportion. None of the skews were correlated with gene length (since there was no correlation between skews and gene length in housekeeping genes, it has not been depicted in the figure). Comparison depicted that gene length influences nucleotide disproportion in pancreatitis genes while in housekeeping genes, it does not. Nucleotide skews have been found to change across the organism's length, and the skew patterns are specific and can be used to classify unknown organisms [27].

### Effect of AT and GC composition of CUB of codons

Generally, the RSCU of AT and GC ending codons are be influenced by AT and GC composition, respectively [28]. To determine the effect of AT and GC composition on AT and GC ending codons in pancreatitis associated genes, we performed a correlation analysis between the RSCU of 59 codons (excluding stop codons, methionine, and tryptophan) and overall AT and GC composition along with AT and GC composition at all the three codon positions. In pancreatitis-associated gene set, AAA, GAA, TCA, GTA, ATA, TTA, TTT, TAT, TGT, ACT, AAT, TGC, TTC, ACC, CCG, ACG, GAG, and GTG codons showed correlation with overall AT and GC composition and AT and GC composition at all the three codon positions. Similarly, in housekeeping genes, CTT, GTT, AAT, GAT, GAA, CTG, AGC, GAC, GAG, and CGC codons correlated with overall AT and GC composition and AT and GC composition at all the three codon positions. AGG (Arg), TCG (Ser), GTC (Val), CGT (Arg), CCA (Pro), and CGA (Arg) were independent of the AT and GC nucleotide composition at all the three codon positions in pancreatitis-associated genes. In the housekeeping genes, only codon AGG had no correlation with overall AT and GC nucleotide composition. At the same time, none of the codons showed independence from AT and GC composition at all the three codon positions. In pancreatitis gene set CGA, CCA, ACT, GTC, AGT, TCT, GGG, CCG, TCG, GCG, and AGG, while in housekeeping gene set CGT, GTC, and GGG codon showed no correlation with ENc. The analysis is suggestive of a clear difference in codon preferences.

### Association of compositional constraint independent codons of pancreatitis associated genes with other parameters

Six codons of pancreatitis associated genes viz. AGG, TCG, GTC, CGT, CCA, and CGA are found to be independent of the influence of compositional constraint. These codons, whether they are affected /influenced by any other parameter or not, were tested by conducting correlation analysis between these six codons and length, CAI (codon adaptation index), ENc (effective number of codons), SCS (scaled chi square), and protein property indices like isoelectric point, instability index, aliphatic index, hydropathicity, grand average of hydropathy (GRAVY), aromaticity (AROMA), and frequency of acidic, basic and neutral amino acids (Table 2). The analysis indicated that, though these codons were free from influence of AT and GC composition, these were still associated with a few of the gene parameters like CAI, CUB, and a few of the protein properties.

**Table 2:** Correlation analysis of codons with various properties of a gene. The table shows the p values. All bold values showed a significant correlation (*p* < 0.05). The italics font showed a negative correlation, while the straight font showed a positive correlation

S. No	Parameters	AGG (Arg)	TCG (Ser)	GTC (Val)	CGT (Arg)	CCA (Pro)	CGA (Arg)
1	length	0.662	*0.416*	*0.085*	0.845	**0.001**	*0.751*
2	CAI	*0.812*	0.319	**0.002**	*0.163*	*0.144*	*0.473*
3	ENc	0.338	*0.704*	*0.169*	**0.039**	0.185	0.110
4	SCS	0.638	*0.995*	*0.225*	0.241	**0.000**	*0.443*
5	PI	*0.094*	*0.211*	***0.012***	0.108	*0.244*	*0.611*
6	Instability Index	0.332	0.110	***0.033***	0.869	**0.049**	0.657
7	Aliphatic Index	*0.417*	*0.770*	0.527	*0.724*	*0.142*	0.543
8	HY	*0.181*	0.802	*0.656*	0.423	*0.064*	0.218
9	Acidic AA	0.244	**0.006**	0.351	*0.080*	0.114	*0.886*
10	Basic AA	*0.242*	0.737	***0.000***	0.181	0.526	*0.335*
11	Neutral AA	0.246	***0.035***	0.088	*0.681*	0.735	*0.613*
12	GRAVY	*0.365*	*0.136*	0.154	0.695	***0.024***	0.462
13	AROMA	*0.639*	0.844	0.052	0.693	*0.289*	0.160

### Neutrality analysis

**Fig. 6:**
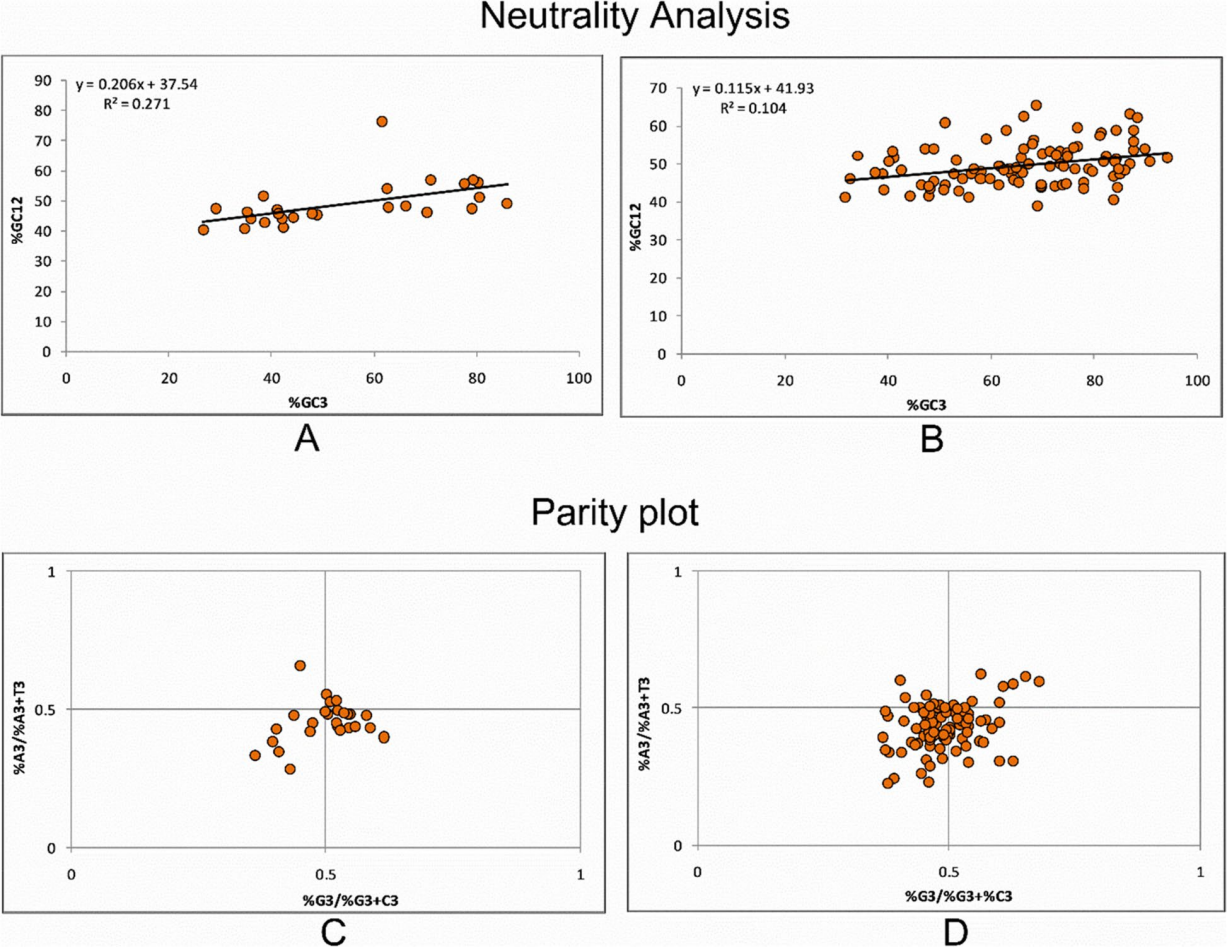
Neutrality analysis for genes: **A** In pancreatitis-associated gene sets, mutational force and selection forces contributed 20.65% and 79.35% in shaping codon usage. **B** In housekeeping genes, mutational force and selection forces contributed 11.5% and 88.5%, respectively, in shaping codon usage. The parity plot analysis **C**. Pancreatitis-associated genes showed a preference for T over A and equal usage of C and G. **D** Housekeeping genes showed a preference for T over A and C over G

A regression plot between %GC3 and %GC12 content shows the equilibrium between the selectional and mutational force [29]. The %GC3 content varied from 26.64% to 85.94%, while %GC12 content varied between 40.28% and 76.31%. The relative neutrality 20.65 indicates that mutational force is attributed to 20.65%. The remaining 79.35% are selectional forces acting on genes related to pancreatitis and suggestive of the dominance of selection force over mutational force (Fig. 6A). Regression analysis for the housekeeping gene showed relative neutrality of 0.115, indicating that mutational force is attributed to 11.5% while selective forces contributed 88.5% (Fig. 6B). In both, the gene sets selection force seems to be dominant; however, selection forces are more on housekeeping genes.

### Parity analysis

Parity analysis shows the preference for purine or pyrimidine at third codon positions. The parity indicates the nucleotide skew at the third codon position. At the center of the plot, A = T, and C = G. A3/A3 + T3 shows the AT bias, while G3/G3 + C3 shows the GC bias at the third codon position. The value of GC bias was 0.497 ± 0.06 and AT bias was 0.4531 ± 0.07 for pancreatitis associated genes. The values show that nucleotides G and C are used almost equally, and among AT pairs, T is preferred over A (Fig. 6C). For housekeeping genes, the value for GC bias at the third codon position was 0.491 ± 0.07, while for AT bias, it was 0.434 ± 0.08. The results suggest the preference of C and T over G and A, respectively (Fig. 6D).

### Effect of mutational force on codon composition

**Fig. 7:**
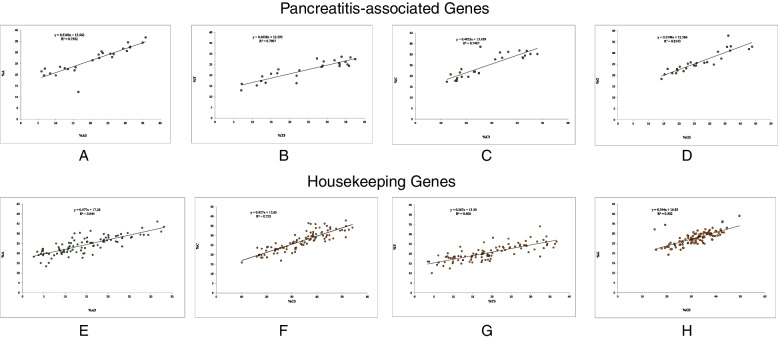
Effects of mutational forces on nucleotide compositions

To determine the effect of mutational force on the nucleotide composition of the gene, a regression analysis was executed between the nucleotide composition at the third codon position and overall nucleotide composition. The analysis revealed that 81.43% of the variation in G nucleotide's overall composition is explained by mutational forces applied on G nucleotide, which is the maximum among all four nucleotides for pancreatitis-associated genes (Fig. 7A, B, C, D). Similarly, a mutation in nucleotides A, T, and C (75.62%, 79.07%, and 74.07%, respectively) also explain the composition of respective nucleotides. In housekeeping genes, mutational forces explained maximum variation in nucleotide C (72.33%) followed by A, T and G nucleotides (67.99%, 60.06% and 50.26%, respectively) (Fig. 7E, F, G, H).

## Discussion

The composition has an essential effect on the codon usage bias of any gene [30]. In the present study mean GC component (50.82%) was slightly higher than AT component (49.17%). However, the difference is more evident in the human alanyl-tRNA synthetase 1 (*AARS*) gene family responsible for producing proteins playing secondary roles in autoimmune myositis. In the alanyl-tRNA synthetase 1 (*AARS)* gene family, the overall percentage of GC (53.76%) content is higher than AT (46.23%). Based on the GC skew, it was evident that G is overrepresented than C at the third codon position. In prokaryotes, the excess of G over C is common and, to a lesser extent, T (over A) in the replication leading strand [25]. GC3 is an imperative indicator of CUB at the third codon position except for Met (AUG) and Trp (UGG) encoding codons [31]. GC content and GC3 components are lower in monocytes than protein-coding genes expressed in B and T lymphocytes and other human protein-coding genes. This variation suggests the role of composition constraint in influencing the codon usage pattern [32]. In the present study, in the pancreatitis-associated genes, G and C are used almost equally, and among AT pairs, T is preferred over A. Different observations are found in the sex determining region of the Y (*SRY)* gene across the mammalian species. In mammalian sex determining region of the Y (*SRY*) gene, C is preferred over G, and A is preferred over T [33]. The genome nucleotide composition variation in GC versus AT is a consequence of interspecies mutation bias difference or action of the selection for different nucleotides or a combination of the two or GC biased gene conversion [34] and a decreasing GC gradient from the 5'- to 3'- ends of coding regions in various organisms have been observed. It results from complex interactions that shape codon composition, especially for efficient energy usage [35]. Therefore, our result indicates a complex bias due to GC bias gene conversion and asymmetrical replication of the leading and lagging strand.

The dinucleotide odds ratio is an indicator of biases in codon usage and sometimes may act as a signature to identify the genetic causes of disease. The dinucleotide odds ratio might indicate horizontal gene transfer [36]. For example, the TpT dinucleotide genotype has been correlated with increased coronary artery disease rates [37]. The odds ratio might be typical of a set of genes. CpG, TpA, and GpT are the dinucleotides with the least odds ratio in the set of 26 genes involved in pancreatitis. CpG and TpA are the dinucleotides that are generally underrepresented in most genes [38]. TpA but not the CpG has adversely affected gene expression [12]. The pattern might be variable for a different set of genes. When we compared the pancreatitis gene set with that of the housekeeping gene, TpA and CpG dinucleotides were found underrepresented in both the gene sets; in the pancreatitis gene set we revealed the underrepresentation of CpG in most of the genes, excluding *CDKN2A* and von Hippel-Lindau tumor suppressor (*VHL)* genes where CpG was overrepresented and in Apolipoprotein A5 (*APOA5)* and Multiple endocrine neoplasia type 1 (*MEN1*) where CpG was randomly used. From Cardon et al. (1994) [39] studies, we might speculate that these genes might have fungal or protest origin. Another speculation is that over usage of CpG might result from a strategy adopted by the cell to attenuate the gene expression [40]. In eukaryotes, CpG and TpA content is depleted because CpG dinucleotides are prone to methylate at the fifth position of cytosine, and subsequent deamination results in the formation of thymidine out of cytosine [41]. In the experiment of Bauer et al. (2010) [42], intragenic CpG content effect on protein expression was observed, and GPP reporter containing CpG depleted versions compared to wild type CpG content had depleted protein expression profile. As per Saxonov et al. (2006) [43], exons are enriched for CpGs compared to introns, and CpGs are also relatively enriched around the transcription start site. The facts mentioned above seem to be correct in our study, where *CDKN2A* and *VHL* genes enriched in CpG dinucleotide were small (399 and 642 base pairs, respectively) and do not contain intronic regions. Overall, CpG content results from a highly dynamic interaction between various factors, including intron/exon length, distance from the promoter, the extent of CpG methylation, and others. Depletion in TpA content is the result of selection since TpA dinucleotide is a part of two out of three stop codons (TAA and TAG) and also reflects instability to nucleolytic cleavage in mRNA [44]. Moreover, TpA is energetically less stable than all other dinucleotides and confers flexibility to the DNA sequence. Avoidance of TpA also is a strategy to avoid inappropriate binding of regulatory factors to TpA containing many regulatory sequences (e.g., TATA box, polyadenylation signals like AATAAA in higher eukaryotes, and TATATA in yeast). The set of genes involved in pancreatitis also is depleted in TpA.

In three dicots, *Glycine max, Arabidopsis thaliana, and Medicago truncatula*, dinucleotides TpG, TpC, GpA, CpA and CpT were over-represented, while CpG and TpA were under-represented [45]. In complete mitochondrial genome study, encompassing 21 species, CpG dinucleotide was under-represented in all animal mitochondria but exhibited variable relative abundance in fungal, protist, and plant mitochondrial genomes [39]. Except for CpG and TpA, in the pancreatitis gene set, GpT was underrepresented, while ApT, GpT, and TpT were underrepresented in the housekeeping gene set. In the present study, CpT, GpA, and TpG were the codons that were not underrepresented in any of the pancreatitis genes envisaged, while TpG, CpA, ApG was not underrepresented in more than 98% of housekeeping genes. TpG is commonly overrepresented dinucleotide across the eukaryotic genome. The same may be explained based on methylation of cytosine in CpG dinucleotide, which results in cytosine to thymidine transition and resultant TpG dinucleotide abundance [46]. Hence no underrepresentation of CpT, and GpA in pancreatitis and CpA, ApG in housekeeping genes suggest dinucleotide frequency as a molecular signature for specific genes. Our observation is supported by the results obtained in the case of the NK2 Homeobox 5 (*NKX*-2.5) gene, which governs heart development in some mammals, where ApT and GpT had the lowest, while CpT and ApG had the highest odds ratio [47].

CTG and GTG codons were overrepresented in the genes involved in pancreatitis. The CTG codon was the most overrepresented in 80.95% of the total 42 genes that were common to primary immunodeficiency and cancer [12]. Contrary to our result, CTG and GTG codons were seldom represented in the Asian tiger mosquito *Aedes albopictus * [48]. Codons containing underrepresented dinucleotides CpG and TpA viz. GTA, TCG, ATA, TTA, CCG, CGT, ACG, GCG, and CTA were underrepresented in the present study, and the results were in concordance with the results of Bordoloi and Nirmala (2021) [49], where similar results were obtained in genes linked with esophagus cancer. Codon CAA was the only exception that was underrepresented and did not contain CpG or TpA dinucleotide. On the other hand, codons CAA and GAA were the codons that were overrepresented in *Triticum aestivum * [50].

Average RSCU values of all C ending codons were between 0.6 to 1.6 and indicated random usage. Amongst T ending codons, all the codons were randomly presented except only codon CGT, which was under-represented. In G ending codons, CpG containing codons were underrepresented, TpG containing codons were overrepresented, and other codons were randomly presented. In pancreatitis and housekeeping gene sets, few codons showed variation in codon usage. Specifically, difference was observed in T ending and G ending codons. On the one hand, GTT is in the pancreatitis gene set; on the other hand, CGT is underrepresented in the housekeeping gene set. Similarly, All C-ending codons are randomly used in pancreatitis, while in housekeeping genes, ATC, GCC, ACC, and AGC codons are overrepresented with random usage of other C-ending codons. We compared all the 59 codons of pancreatitis and housekeeping gene set with 1000 times permutation. We observed that out of 59 codons, 32 codons were significantly different in pancreatitis and housekeeping gene sets. In another study by Chakraborty et al., 2020 [51], 11 codons significantly differed between obesity and housekeeping genes. AGG, CGC, ATT, and CGA for pancreatitis-associated genes, while CGT, AGG, AGC, and CTG for housekeeping genes contributed the maximum to codon bias.

Frequency of one codon for housekeeping genes (AUA-Ile) (Fig. 4A) and five codons for pancreatitis-associated genes (ACG-Thr, CGT-Arg, TCG-Ser, CCG-Pro, GCG-Ala) (Fig. 4B) was found below 0.5%. The presence of rare codon reduce the translation rate by causing ribosome stalling and, therefore, may be helping in fine-tuning translation rates [52] and poorly expressing genes prefer rare codons [53]. Overall comparison between pancreatitis and housekeeping gene indicated a different codon usage pattern based on different codon choices, codons influencing the bias the most, rare codons, and abundant codon pairs. Studies have suggested numerous factors affecting codon usage bias, including GC-content [54], gene size [55], gene expression level [56] and gene recombination rate [57], gene expression level, gene length, gene translation initiation signal, protein amino acid composition, protein structure, tRNA abundance, mutation frequency and patterns, and GC compositions [58], intron length [59] the aromaticity [60] and the hydrophobicity [61], aliphatic index of protein [62], etc. There is a strong negative correlation between codon usage and protein length in distantly related multicellular eukaryotes (*Caenorhabditis elegans*, *Drosophila melanogaster*, and *Arabidopsis thaliana*), and this effect is not due to the higher protein expression level of shorter genes. However, selection pressure is low on longer genes than shorter ones [55]. The results concordance with the present study results and suggest selectional force operative in pancreatitis-associated genes. In mammalian lineages, asymmetry in the frequency of nucleotide substitution in leading and lagging strands is demonstrated, resulting in asymmetry in nucleotide content in most genes [63]. GC skew is commonly employed to identify the origin of DNA replication in prokaryotes. Out of six nucleotide skews (AT skews, GC skews, purine skew, pyrimidine skew, keto skew, and amino skews) studied in the present study, purine skew, pyrimidine skew, keto skew, and amino skews were found positively correlated with the length of the gene. It indicated that these four nucleotide disproportion indices increase with an increase in length. Contrary to pancreatitis-associated genes, housekeeping genes do not show a correlation between nucleotide disproportion indices and gene length. The results again suggest selective forces acting on pancreatitis-associated genes where an enhancement in gene length results in increased nucleotide disproportion [25]. Compositional features are essential in molecular studies of any gene. Using the gene compositional features and gene expression profile, a model has been developed by Elhaik and colleagues to predict gene methylation in *O. Sativa* genes [64]. Eventually, DNA base composition can modulate the epigenome and, ultimately, gene expression [65]. In the present study, we found a significant association between GC3 and CAI, which is indicative of the role of mutational bias on gene expression. Our observation contradicts the findings of Halder et al. (2017) [66], who found GC content as not a good predictor of human gene expression based on data derived from 40 genes. We found a positive association between CUB and GC composition at GC1 and GC2 positions but not at GC3. Our data is in concordance with Mazumder et al., 2019 [23], who found a highly significant association between CUB and GC1 and GC2.

The GC-content of organisms is a highly variable feature and ranges from lower than 25% to higher than 75% [67]. Higher GC content suggests higher usage of GC ending codons and vice versa [68]. In the present study, codons AGG (Arg), TCG (Ser), GTC (Val) were independent of the GC, while CGT (Arg), CCA (Pro), and CGA (Arg) were independent of the AT nucleotide composition at all the three codon positions. These codons contributed very little to PC1 and PC2 in PC analysis. The high content of GC ending codons is present in disorder-promoting amino acids in intrinsically disordered regions of proteins. Intrinsically disordered regions (IDRs) are protein regions prone to inefficient folding and display variable confirmations throughout evolution and the population [69]. Among six codons independent of GC or AT content, four accounts for Arginine and Proline. Also, all these four codons showed RSCU values from complete absence (RSCU value 0) to overrepresentation (RSCU value ≥ 1.6), indicating a specific kind of selection acting on these codons to meet the requirements of intrinsically disordered regions of specific proteins. Proline and arginine knew to be disorder-promoting residues [70]; hence it can be speculated that independence of compositional constrain is a result of high order selection force. These nucleotide compositions independent codons are how influenced by other factors were envisaged by correlation analysis between these codons and length, CAI, ENc, SCS, and protein property indices like isoelectric point, instability index, aliphatic index, hydropathicity, GRAVY, AROMA, and frequency of acidic, basic and neutral amino acids. CCA encoding for proline was the codon that positively correlated with length and CUB. Codon encoding for valine (GTC) had a positive relationship with gene expression, and CGT (Arg) also had a positive association with CUB. This association indicated that though these codons are independent of nucleotide composition but have a significant association with length, and CUB.

CAI measures synonymous codon usage bias towards optimal codons in highly expressed genes. High CAI is suggestive of a high gene expression level [71] and is often used to optimize heterologous expression [72]. CAI had a negative association with CUB and gene length in the present work, while positive with GC3. Length was negatively correlated with CAI in the pancreatitis associated genes; however, the same is not valid for each set of genes. In peramine-coding genes had no association with gene expression level or GC content [73], and the similar result was obtained with housekeeping genes in current study. SCS ranged between 0.01 and 0.6 in the present study and indicated low to moderate bias. Similar to our case, SCS for Major histocompatibility (MHC) genes also is low, with SCS 0.22 for chimpanzees MHC and 0.34 for humans. Major Histocompatibility Complex (HLA) class II beta chain genes exhibit comparatively moderate to high CUB bias (0.53) [74]. A neutrality plot indicates equilibrium between the selection and mutational force [75]. In the present study, we had a slope of the regression line less than 0.5, indicating the dominance of selection pressure. The selectional force was 20.35%, while the mutational force was attributed to 79.65%. Similar results were obtained by Uddin et al. (2020) [75], who also found dominance of selection pressure in shaping codon usage in *ATP6* and *ATP8* genes of fishes, aves, and mammals.

To understand the effects of mutational force on composition, we performed regression analysis and found that mutational force significantly played a role in deciding the compositional constraints. Mutational dynamics is often helpful in analyzing both base composition and codon usage bias. Silent sites in coding sequences in cpDNA appear to be at equilibrium of selection and mutation, while noncoding has a significantly lower A + T content. It suggests that mutational dynamics are complex and must be evaluated for individual species [76]. The mutation plays a significant role in all the nucleotide compositions in the present study. The effect was a maximum for nucleotide G, where 81.43% of mutations explain the composition of nucleotide G. On the other hand, in housekeeping genes, the effects of mutational forces were maximum in deciding the composition of nucleotide C (72.33%). Furthermore, both gene sets use different rare codons, and; GAA-GAA codon pair and GAG-GAG codon pair were most frequent in pancreatitis and housekeeping associated gene sets, respectively. Based on these evidences, it can be said that the pancreatitis-associated gene set exhibits a specific codon usage pattern.

## Conclusions

The present study envisages the molecular characteristics and features associated with codon usage. Compositional analysis of 26 genes envisaged in our study indicated almost equal AT and GC components usage. Among GC, both the G and C components were used equally, while in AT pair T is preferred over A based on skew analysis, owing to the possible role of mutational forces in replicatory leading strand. The dinucleotide odds ratio, suggestive of molecular signature, revealed CpG and TpA, (generally underrepresented in the mammalian genome), and GpT to have the least odds ratio. CTG and GTG codons were overrepresented in the set of genes involved in pancreatitis owing to the overabundance of TpG dinucleotides. Here GpT despite being part of the GTG codon, which is an abundant codon, is underrepresented, suggestive of selectional forces acting on GpT dinucleotide. A negative association between codon usage and protein length has been observed and underscores the importance of selection force. Purine, pyrimidine, keto, and amino skews had a significantly positive association with the length of the gene. The same indicated that the nucleotide disproportion increased proportionally with the increasing length. SCS, ENc and PCA analysis indicated the lower CUB in pancreatitis-associated genes.

Synonymous codon variants are responsible for causing ailments through alteration to various molecular properties of a gene, including the nucleotide skews, DNA and mRNA stability, composition at various codon positions, and rate and amplitude of gene expression. A comparative analysis between pancreatitis and housekeeping associated gene sets, revealed that codon usage pattern is distinct for pancreatitis associated gene set as evidenced by variance analysis, PCA analysis and comparison of rare codon and abundant codon pairs. All observations will be helpful in knowing various evolutionary forces acting on gene sets involved in pancreatitis and provide insight into the silent changes in the nucleotide sequence, which is a possible cause of ailments.

## Methods

### Sequence retrieval

Various commercial and academic institutions offer genetic testing for pancreatitis. Different genes with variation in numbers and in the genes itself are used in panels used for diagnosis. In Genetic Testing registry (GTR), National Center for Biotechnology Information (NCBI), many such gene panels are available and out of many, we chose a panel of 26 gene sequences available for commercial diagnosis for pancreatitis, offered by LifeLabs Genetics, 175 Galaxy Blvd Suite 105, Etobicoke, ON M9W 5R8, Canada, which is using maximum numbers of genes for pancreatitis testing. Hence to make out test statistically maximum significant we took the gene panel offered by LifeLabs Genetics. After obtaining the names of genes, the sequences were retrieved from NCBI nucleotide. For comparative analysis randomly selected 98 housekeeping gene sequences were also obtained from NCBI. All the sequences were qualified based on the gene sequence in multiples of three nucleotides, no redundant nucleotides, and no stop codon in between. The selection criteria for both the pancreatitis associated and housekeeping genes were kept similar for both the gene sets. Accession numbers of the sequences used in the study are given in supplementary table 1.

### Nucleotide composition

The nucleotide composition of each gene was determined with nucleotide compositions at all three positions of codons. GC composition at first and second codon position (%GC12) and %GC3 were used to construct a neutrality plot indicative of equilibrium between mutational and selection forces. The percent composition of all the four nucleotides at third codon positions %A3, %T3, %G3, and %C3 were used in constructing the parity plot. Other compositional parameters were used for various other studies. A total of 20 compositional parameters (overall percent composition of nucleotide A, T, C and G (%A, %T, %C, %G), percent composition of nucleotides at first codon position (%A1, %T1, %C1, %G1), percent composition of nucleotides at second codon position (%A2, %T2, %C2, %G2), percent composition of nucleotides at third codon position (%A3, %T3, %C3, % G3), overall percent GC composition and composition at first, second and third position (%GC, %GC1, %GC2, %GC3) were envisaged for the study).

### Odds ratio

The frequency of the dinucleotide features is critical as it might affect the usage of codons [17]. The dinucleotide frequency indicates usage of the favorable or unfavorable nucleotide pairs and is indicative of both the selectional and mutational forces [62]. The odds ratio is calculated as observed to the expected frequency of a dinucleotide and is a binding force responsible for shaping codon pair bias. The odds ratio ≤ 0.78 and ≥ 1.23 indicated dinucleotide underrepresentation and overrepresentation, respectively [40].

### Synonymous codon usage analyses (RSCU)

The RSCU value indicates how efficiently one synonymous codon is used over others for a single amino acid. Higher RSCU value indicates overuse of that codon while the lower values indicate vice versa. The RSCU value for a codon is the observed frequency divided by the expected frequency when all the synonymous codons for an amino acid are equally used [77]. The RSCU values less than 0.6 are considered underrepresented, while values above 1.6 are considered over-represented [78].

### Codon adaptation index (CAI)

CAI is one of the measures to determine the difference in the synonymous codon frequency in a given transcript. This CAI helps to understand the gene expression and elucidate the molecular mechanism for gene evolution [50, 79]. CAI is a popular numerical estimator to predict the gene expressivity and estimation of highly expressed genes [80]. Natural selection is a driving force that chooses some codons over the others. CAI value is calculated using the highly expressed genes as reference set [77]and it helps in estimating the strength of translational selection and hence allows prediction of gene expression level based on RACU values of codons. In present study the CAI values of 26 genes were calculated using the software developed by Bourret et al., 2019 [81]. For calculation of CAI value human codon usage table was used as reference set available at Kazusa codon usage database.

CAI values of different pancreatitis-associated and housekeeping genes envisaged in the present study are given in Table 3.

**Table 3:** CAI value of various pancreatitis associated and housekeeping genes

	GENE	CAI	GENE	CAI	GENE	CAI
Pancreatitis genes	*APC*	0.699	*CFTR*	0.694	*PALB2*	0.697
	*APOA5*	0.83	*CPA1*	0.839	*PMS2*	0.74
	*APOC2*	0.759	*CTRC*	0.836	*PRSS1*	0.829
	*ATM*	0.675	*EPCAM*	0.712	*SMAD4*	0.712
	*BMPR1A*	0.711	*GPIHBP1*	0.842	*SPINK1*	0.734
	*BRCA1*	0.715	*MEN1*	0.823	*STK11*	0.835
	*BRCA2*	0.691	*MLH1*	0.751	*TP53*	0.798
	*CASR*	0.808	*MSH2*	0.694	*VHL*	0.77
	*CDKN2A*	0.676	*MSH6*	0.716	-	-
Housekeeping genes	*AKAP9*	0.707	*ACAD9*	0.806	*FLNA*	0.84
	*ABCD3*	0.711	*DDB1*	0.81	*UBC*	0.842
	*ABCB7*	0.715	*CD63*	0.81	*SCYL1*	0.843
	*ZFR*	0.719	*JAGN1*	0.811	*ALDOA*	0.844
	*CTNNB1*	0.722	*BCAP31*	0.811	*PTOV1*	0.847
	*AGPS*	0.724	*HAGH*	0.812	*CSTB*	0.847
	*ALG8*	0.726	*ALAD*	0.813	*IRAK1*	0.849
	*PABPC1*	0.726	*HDAC1*	0.813	*AHCY*	0.85
	*DLG1*	0.727	*YY1*	0.814	*BSG*	0.85
	*LDHA*	0.728	*PYCR2*	0.817	*PURA*	0.85
	*COPA*	0.78	*AKAP8*	0.818	*JUP*	0.853
	*PGK1*	0.78	*RPL11*	0.819	*RPL19*	0.853
	*HNRNPA1*	0.78	*ABCF1*	0.82	*CTSD*	0.856
	*MGP*	0.782	*DAG1*	0.82	*INF2*	0.857
	*MLH1*	0.782	*CTTN*	0.822	*AIP*	0.862
	*ACOX1*	0.784	*VIM*	0.822	*CHST12*	0.862
	*AFF4*	0.787	*TAB1*	0.823	*COMT*	0.864
	*FECH*	0.787	*ARAF*	0.823	*CD151*	0.865
	*RPS27A*	0.787	*AK2*	0.825	*ADIPOR1*	0.867
	*AAAS*	0.788	*PKD1*	0.826	*STUB1*	0.868
	*GSTO1*	0.789	*KAT5*	0.827	*CD81*	0.874
	*LARS2*	0.79	*SERPINA3*	0.827	*AKT1*	0.876
	*FUS*	0.79	*HSPB1*	0.829	*UBTF*	0.877
	*GCLC*	0.791	*CIC*	0.83	*ACTG1*	0.886
	*ACVR1B*	0.793	*FIBP*	0.83	*BTG2*	0.886
	*INTS3*	0.793	*CCR9*	0.831	*ACTB*	0.897
	*NPC2*	0.793	*FTSJ1*	0.832	*DDX17*	0.742
	*B2M*	0.793	*CCND2*	0.833	*TXLNG*	0.75
	*ILK*	0.795	*GALNS*	0.835	*CDK13*	0.758
	*CHST7*	0.8	*SIL1*	0.835	*ACOT9*	0.761
	*ELAC2*	0.8	*POLR1C*	0.837	*ACBD3*	0.768
	*ZXDA*	0.802	*NCOR2*	0.837	*RUFY1*	0.774
	*NDUFA4*	0.804	*CLU*	0.837	-	-

### Scaled chi-square (SCS) and effective number of codons (ENc)

Various measures of codon usage bias (CUB), both directional and non-directional, have been developed. The present study determined the directional measure SCS [82] and the non-directional measure adequate number of codons ENc [83]. SCS is a deviation from equal usage of synonymous codons divided by total codons, excluding Trp, Met, and termination codons. The values for the genes under study were calculated using the software developed by Bourret et al., (2019) [81]. SCS value ranges between 0 and 1, and higher values show higher bias [84]. ENc values range between 20 and 61, and low values indicate higher bias while higher indicate lower bias. ENc is less sensitive than SCS when the gene length is considered [84].

### Nucleotide skews

Nucleotide skew is a phenomenon present across the genomes and is the measure of nucleotide disproportion [85]. A deviation from the PR2 rule indicates the role of selectional and mutational forces in the DNA duplex and as a result, stands bias is generated. The skews in a strand may be calculated with the formula XY skew = (X–Y)/X + Y), where X and Y are the complementary nucleotides [86]. The skews we used in the present study are GC skew (G and C), AT skew (A and T), purine skews (G and A), pyrimidine skew (C and T), keto skew (G and T), and amino skew (A and C) [87].

### Statistical analysis

Correlation analysis, partial least squares regression, F test and principal component analysis were carried out using PAST4 statistical software.

## Supplementary Information

**Additional file 1:**

## Data Availability

All data generated or analysed during the study is included in this published article and its supplementary information files.

## Abbreviations

%A, %T, %G, %G: Overall percent composition of A, T, C and G nucleotides
%A1, %T1, %C1, %G1: Percent composition of A, T, C and G nucleotides at first codon position
%A2, %T2, %C2, %G2: Percent composition of A, T, C and G nucleotides at second codon position
%A3, %T3, %C3, % G3: Percent composition of A, T, C and G nucleotides at third codon position
%GC, %GC1, %GC2, %: Overall percent GC composition and composition at first, second and third position
AARS: Alanyl-tRNA synthetase 1
ADAMTS13: ADAM Metallopeptidase With Thrombospondin Type 1 Motif 13
APOA5: Apolipoprotein A5
AROMA: Aromaticity
CAI: Codon adaptation index
CASR: Calcium Sensing Receptor
CDH23: Cadherin Related 23
CDKN2A: Cyclin-dependent kinase inhibitor 2A
CEL: Carboxyl Ester Lipase
CFTR: Transmembrane Conductance Regulator
CLDN2: Claudin 2
CPA1: Carboxypeptidase A1
CTRC: Chymotrypsin C
CTSB: Cathepsin B
CUB: Codon usage bias
ENc: Effective number of codons
FUT2: Fucosyltransferase 2
GRAVY: Grand average of hydropathy
HLA: Major Histocompatibility Complex
IDRs: Intrinsically disordered regions
ITIH2: Inter-Alpha-Trypsin Inhibitor Heavy Chain 2
MEN1: Multiple endocrine neoplasia type 1
MHC: Major histocompatibility
MYO9B: Myosin IXB
PC1 and PC2: Principal Component 1 and 2
PRSS1: Serine Protease 1
RHBDD2: Rhomboid Domain Containing2
RSCU: Relative synonymous codon usage
SCS: Scaled Chi Square
SLC9A3R1: SLC9A3 Regulator 1
SPINK1: Serine protease inhibitor Kazal type 1
SRY: Sex determining region of the Y
sSNV: Synonymous single nucleotide variants
UBR1: Ubiquitin Protein Ligase E3 Component N-Recognin 1
VHL: Von Hippel-Lindau Tumor Suppressor
VWF: Von Willebrand Factor
